# Immune Thrombocytopenia in a Child with T Cell Lymphoblastic Lymphoma

**DOI:** 10.1155/2016/2897325

**Published:** 2016-09-07

**Authors:** Kayo Tokeji, Sachi Sakaguchi, Tomoko Kurimoto, Junya Fujimura, Toshiaki Shimizu

**Affiliations:** Department of Pediatrics, Faculty of Medicine, Juntendo University, Tokyo, Japan

## Abstract

We describe the case of a 13-year-old boy who presented with persistent thrombocytopenia during maintenance chemotherapy with mercaptopurine and methotrexate for T cell lymphoblastic lymphoma. He was diagnosed with immune thrombocytopenia (ITP) after thorough investigations for the relapse of lymphoma and was successfully treated with immunoglobulin and steroids. ITP is known to be associated with chronic lymphocytic leukemia, Hodgkin lymphoma, and various types of non-Hodgkin lymphoma but rarely with T cell non-Hodgkin lymphoma or in children. Diagnosis of ITP with lymphoma is challenging due to the many factors affecting platelet counts, and ITP often complicates the diagnosis or treatment course of lymphoma. The underlying mechanism of ITP with NHL is still unclear. Drug-induced immunomodulation with a reduction of regulatory T cells might have contributed to the development of ITP in our case.

## 1. Introduction

Immune thrombocytopenia (ITP) is one of the most common causes of symptomatic thrombocytopenia in children. Secondary ITP attributed to coexisting conditions such as chronic lymphocytic leukemia, Hodgkin lymphoma, and various types of non-Hodgkin lymphoma (NHL) have been reported in adults but rarely in children. Here, we describe a case of a 13-year-old boy who was diagnosed with ITP during maintenance chemotherapy for T cell lymphoblastic lymphoma (T-LBL).

## 2. Case Presentation

A 13-year-old Japanese boy was referred to our hospital complaining of a right inguinal mass. Biopsy of the mass confirmed an enlarged inguinal lymph node with monotonous proliferation of medium- to large-sized atypical lymphoid cells positive for CD2, cytoplasmic CD3, CD5, and CD7 but negative for CD10, CD19, CD20, and CD22. Although his initial blood examination revealed normal white blood cells (WBCs, 4,500/*μ*L), hemoglobin (14.7 g/dL), and platelets (456,000/*μ*L), bone marrow examination revealed infiltration of atypical blastic cells, and thus he was diagnosed with T-LBL stage IV. Complete remission was achieved after induction chemotherapy. Subsequent courses of chemotherapy were uneventful, and he started maintenance therapy with daily mercaptopurine (60 mg/m^2^) and weekly methotrexate (25 mg/m^2^). The first 8 weeks of maintenance therapy was uneventful. Complete blood cell counts at the clinic visit on the fifth week were within expected ranges for WBCs (2,300/*μ*L), hemoglobin (11.1 g/dL), and platelets (125,000/*μ*L).

Four weeks later, at the ninth week clinic visit, the patient presented with petechiae on his lower limbs. Blood examination revealed low platelet counts (37,000/*μ*L) and normal hemoglobin levels (11.1 g/dL). Also, as expected, WBC counts were low (1,400/*μ*L; neutrophils 32.7%, lymphocytes 40.4%, and blastic cells 0%) due to maintenance therapy. His blood chemistry was normal. His physical examination was unremarkable except for the petechiae. Maintenance therapy was suspended, but thrombocytopenia persisted with low platelet counts (7,000–25,000/*μ*L). Bone marrow aspiration and biopsy revealed normocellular bone marrow, normal megakaryocyte counts (6/*μ*L), and no lymphoma cell infiltration. A whole-body ^18^F-FDG positron emission tomography-computed tomography scan was negative. Platelet-associated immunoglobulin G (PAIgG) was high (446 ng/10^7^ cells; normal range: 9–25 ng/10^7^ cells). The patient received multiple platelet transfusions. Platelet counts immediately after the transfusions were lower than estimated counts, indicating an increase in platelet destruction rather than a decrease of platelet production.

Based on these findings, the patient was diagnosed with ITP, and intravenous immunoglobulin (IVIG; 1 g/kg) was administered 7 weeks after the discontinuation of maintenance therapy. Five days after IVIG therapy, his platelet count recovered (to 100,000/*μ*L), and he was started on oral prednisolone (1 mg/kg/day). Maintenance chemotherapy was resumed 3 weeks after IVIG therapy. Prednisolone was gradually decreased and discontinued 11 weeks after IVIG therapy. The patient completed the 74-week maintenance chemotherapy and has been off therapy for more than 4 months with normal platelet counts (200,000–300,000/*μ*L).

## 3. Discussion

ITP can present before, at, or after the diagnosis of NHL and often complicates the disease course of NHL. The reported prevalence of ITP in NHL is 0.76% based on four studies including 1,850 patients [1]. A cohort study using a Swedish nation-wide database showed an increased incidence ratio of 7.5 for NHL after diagnosis of ITP [2]. Among the various types of NHL reported in association with ITP, T cell lymphoma seems to be rare. A literature search using PubMed through December 2015 with the MeSH terms of “lymphoma, non-hodgkin” and “purpura, thrombocytopenic, idiopathic” identified 13 cases of ITP in association with T cell lymphoma, including 12 cases of angioimmunoblastic T cell lymphoma and one case of diffuse large T cell lymphoma. The patients with angioimmunoblastic T cell lymphoma were all Japanese, between 56 and 88 years of age, with increased levels of PAIgG [3]. The patient with diffuse large T cell lymphoma was a 53-year-old woman who was diagnosed with chronic ITP 3 years prior to the diagnosis of lymphoma [4].

The underlying mechanism of ITP with NHL is still unclear. Increased antiplatelet antibodies were observed in most cases of ITP with NHL. As several studies have shown the role of platelet-specific autoreactive T cells in primary ITP [5], it is possible that T cell origin lymphoma cells activate B cells to produce antiplatelet antibodies. This may explain why ITP with NHL is often resistant to standard therapies such as steroids or IVIG but is resolved by treatment for NHL. However, this is less likely to explain the development of ITP in our patient, as he maintained complete remission of lymphoma at the onset of ITP and showed good responses to IVIG and steroid therapy. Drug-induced immunomodulation may contribute to the development of ITP in patients undergoing chemotherapy. Liu et al. reported that CD4^+^CD25^+^ regulatory T cells are reduced in number and function in ITP patients [5]. In our patient, ITP developed during maintenance therapy with mercaptopurine and methotrexate, which causes prolonged depletion of CD4^+^ cells. Genetic susceptibility predisposing to both ITP and NHL may be another potential mechanism.

In summary, we describe the case of a 13-year-old boy diagnosed with ITP during maintenance chemotherapy for T-LBL. Diagnosis of ITP with NHL is often challenging due to many factors affecting platelet counts. ITP is associated with various types of NHL in adults but rarely with T cell lymphoma. The underlying mechanisms of the development of ITP with NHL and factors influencing treatment responses remain to be explored.
